# Association between Two Common Polymorphisms and Risk of Hepatocellular Carcinoma: Evidence from an Updated Meta-Analysis

**DOI:** 10.1155/2014/468605

**Published:** 2014-04-17

**Authors:** Zhaoming Wang, Lei Zhang, Xuesong Shi, Huayu Xu, Ting Wang, Jianmin Bian

**Affiliations:** ^1^Department of General Surgery, The Affiliated Nanjing First Hospital with Nanjing Medical University, No. 68 Changle Road, Nanjing 210006, China; ^2^Department of General Surgery, The First People's Hospital of Changzhou, Changzhou 213000, China; ^3^The First Clinical Medical College, Nanjing Medical University, Nanjing 210000, China; ^4^School of Public Health, Nanjing Medical University, Nanjing 210000, China

## Abstract

*Background*. Recent studies suggested that two common polymorphisms, miR-146a G>C and miR-196a2 C>T, may be associated with individual susceptibility to hepatocellular carcinoma (HCC). However, the results remain conflicting rather than conclusive. *Object*. The aim of this study was to assess the association between miR-146a G>C and miR-196a2 C>T polymorphisms and the risk of HCC. *Methods*. A meta-analysis of 17 studies (10938 cases and 11967 controls) was performed. Odds ratios and 95% confidence intervals were used to evaluate the strength of the association. *Results*. For miR-146a G>C, the variant genotypes were associated with a decreased risk of HCC (CC versus GG: OR = 0.780 and 95% CI 0.700–0.869; GC/CC versus GG: OR = 0.865 and 95% CI 0.787–0.952; CC versus GC/GG: OR = 0.835 and 95% CI 0.774–0.901). For miR-196a2 C>T, significant association was also observed (TT versus CC: OR = 0.783, 95% CI: 0.649–0.943, and *P* = 0.010; CT versus CC: OR = 0.831, 95% CI 0.714–0.967, and *P* = 0.017; CT/TT versus CC: OR = 0.817, 95% CI 0.703–0.949, and *P* = 0.008). *Conclusion*. The two common polymorphisms miR-146a G>C and miR-196a2 C>T were associated with decreased HCC susceptibility, especially in Asian population.

## 1. Introduction

Liver cancer is the sixth most common cancer in the world, with 782,000 new cases diagnosed in 2012. Hepatocellular carcinoma (HCC) is its dominant histological type and accounts for 70–85% of primary liver cancer [1]. Because of the high fatality rates, its incidence approximately equals the mortality rate and nearly 53% of all liver cancer deaths worldwide were in China [2, 3]. Chronic hepatitis B virus (HBV) or hepatitis C virus (HCV) infections are the major cause of HCC, but only a fraction of infected patients develop HCC during their lifetime [4, 5]. Recent studies have demonstrated that genetic alterations may be involved in the development and prognosis of HCC [6, 7].

MicroRNAs (miRNAs) are a large family of short noncoding and evolutionarily conserved RNAs (about 21–23 nucleotides) that function as negative gene regulators [8]. They exert their regulatory effects by binding to the 3′ untranslated region of target messenger RNAs (mRNAs) imperfectly, repressing target gene expression at a posttranscriptional level and inducing mRNA degradation eventually. These small molecules have been shown to play an important role in malignancy by targeting various tumor suppressors and oncogenes, taking part in cancer stem cell biology, angiogenesis, and epithelial-mesenchymal transition [9–12]. Single nucleotide polymorphism (SNP) is the most common genetic variation. SNPs in miRNA may affect the expression and function of mature miRNA and thereby influence individual susceptibility to cancer [13–15]. SNPs miR-146a G>C (rs2910164) and miR-196a2 C>T (rs11614913) are two of the most popular miRNA polymorphisms and have been shown to relate to tumorigenesis in several studies [16–20].

To date, several studies have investigated the association between the two polymorphisms miR-146a G>C and miR-196a2 C>T and hepatocellular carcinoma susceptibility. However, the results remain inconsistent rather than conclusive. In order to estimate the overall risk of miR-146a G>C and miR-196a2 C>T polymorphisms associated with hepatocellular carcinoma and to quantify the potential between study heterogeneity, we carried out a meta-analysis on all eligible case-control studies with a total of 10938 hepatocellular carcinoma cases and 11967 controls.

## 2. Materials and Methods

### 2.1. Identification and Eligibility of Relevant Studies

We searched the electronic literature from PubMed, EMBASE, Cochrane Central Register of Controlled Trials, ScienceDirect, Chinese National Knowledge Infrastructure (CNKI) databases, and Wanfang databases for all relevant reports (the last search update was February 10, 2014), using the search terms “miR-196a2 or microrna 196a2 or rs11614913 or miR-146a or microrna 146a or rs2910164,” “polymorphism or variant or SNP,” and “hepatocellular carcinoma or liver cancer or HCC.” Publication country and publication language were not restricted in our search. In addition, studies were identified by a manual search of the reference lists of original studies. Of the studies with the same or overlapping data published by the same investigators, the most recent or complete articles with the largest sample sizes were included. In our meta-analysis, studies had to meet the following criteria: (a) evaluated the correlation between SNPs miR-146a rs2910164 and/or miR-196a2 rs11614913 and susceptibility to hepatocellular carcinoma, (b) contained available genotype frequency for both cases and controls, and (c) used a case-control design. Studies were mainly excluded for the following reasons: (a) no control population, (b) duplicating the previous publication, and (c) not for human cancer research.

### 2.2. Data Extraction

Two of the authors (Zhaoming Wang and Lei Zhang) extracted data independently complying with the inclusion criteria after the concealment of titles, authors, journals, supporting organizations, and funds to avoid investigators' bias. In the present study, the following variables were collected for each study: the first author's last name, year of publication, country of origin, ethnicity, source of controls (population- or hospital-based controls), genotyping method, and sample sizes of genotyped cases and controls. In the cases of conflicting evaluation, the two investigators checked the data and agreement was reached after a discussion. If disagreement still existed, senior investigator Jianmin Bian was invited to the discussion.

### 2.3. Statistical Analysis

For the control group of each study, the Hardy-Weinberg equilibrium (HWE) was calculated using a goodness-of-fit chi-square test. Odds ratios (ORs) with 95% confidence intervals (CIs) were used to assess the strength of association between the miR-146a rs2910164 and miR-196a2 rs11614913 polymorphism and susceptibility to HCC. The pooled ORs were performed for allele frequency comparison (miR-146a G>C: C versus G, and miR-196a2 C>T: T versus C), codominant model (miR-146a G>C: GC versus GG, CC versus GG, miR-196a2 C>T: CT versus CC, and TT versus CC), dominant model (miR-146a G>C: GC/CC versus GG, and miR-196a2 C>T: CT/TT versus CC), and recessive model (miR-146a G>C: CC versus GC/GG, and miR-196a2 C>T: TT versus CT/CC), respectively. The significance of pooled ORs was determined by *Z*-test and *P* < 0.05 was considered as statistically significant. The heterogeneity between the studies was assessed by Cochran's *Q*-test [21]. If the studies were shown to be homogeneous with a *P* > 0.10 for the *Q* test, the summary of OR estimate of each study was calculated using a fixed-effects model (the Mantel-Haenszel method) [22]. Otherwise, the random-effects model (the DerSimonian and Laird method) was used [23]. Sensitivity analyses were also performed to assess the stability of the results by deleting a single study in the meta-analysis each time to reflect the influence of the individual data set to the summary OR. To test the publication bias, both Funnel plots and Egger's linear regression tests were used [24]. All analyses were performed with Stata software (version 10.0; StataCorp LP, College Station, TX), using two-sided *P* values.

## 3. Results

### 3.1. Characteristics of Studies

**Figure 1:**
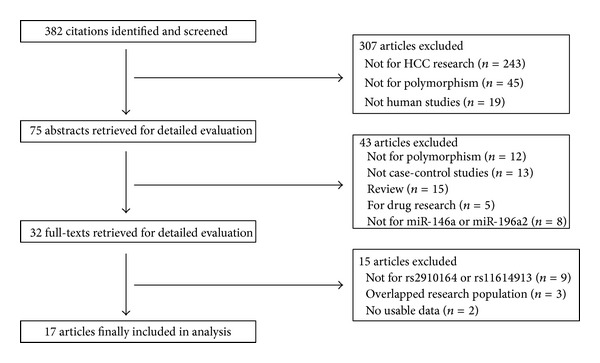
Articles identified with criteria for inclusion and exclusion.

There were 382 published articles relevant to the search terms (Figure 1). By choosing additional filters, 307 of these papers were excluded (243 not for hepatocellular carcinoma research, 45 not for polymorphism, and 19 not for human studies). 43 of these studies were excluded by screening the titles and abstracts. Only 32 articles were left for full text review, and among them another 15 were excluded. Finally, a total of 17 eligible studies involving 5689 cases and 6790 controls for miR-146a G>C and 10 studies involving 5249 cases and 5177 controls for miR-196a2 C>T were included in this meta-analysis [25–41]. The characteristics of the selected studies are summarized in Table 1. For miR-146a G>C, there were 12 studies on Asian population (11 Chinese and 1 Korean) and 1 study on Caucasian population (Turkish). As for miR-196a2 C>T, 9 studies were carried out on Asians (8 Chinese and 1 Korean) and one study on Caucasian (Turkish). Hepatocellular carcinomas were confirmed histologically or pathologically in most studies. All of the controls were matched with respect to ethnicity. Among them, 16 studies were population based while one was hospital based. Several genotyping methods were used in the studies, including polymerase chain reaction-restriction fragment length polymorphism (PCR-RFLP), primer introduced restriction analysis-PCR (PIRA-PCR), PCR-ligase detection reaction (PCR-LDR), allele specific-PCR (AS-PCR), matrix-assisted laser desorption/ionization time-of-flight (MALDI-TOF), and MassArray. The distribution of genotypes in the controls was in agreement with HWE in all studies.

### 3.2. Quantitative Synthesis

**Figure 2:**
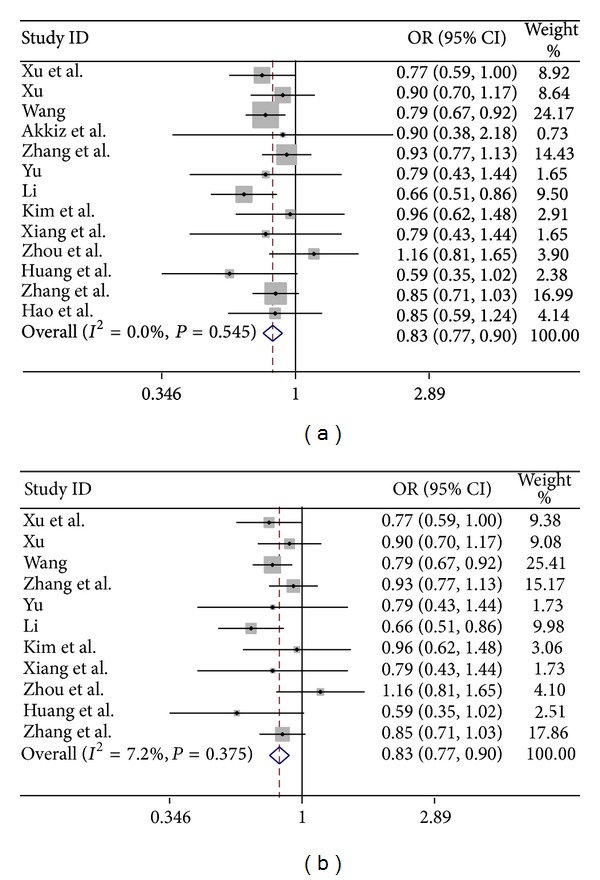
Forest plot of hepatocellular carcinoma risk associated with the* mir-146a G>C* polymorphism (CC versus GC/GG) in overall population (a) and in Asian population (b).

As shown in Table 2, the miR-146a G>C polymorphism was significantly associated with a decreased risk of hepatocellular carcinoma in the following models: C versus G: OR = 0.883, 95% CI 0.839–0.930, and *P* = 0.000; CC versus GG: OR = 0.780, 95% CI 0.700–0.869, and *P* = 0.000; GC/CC versus GG: OR = 0.865, 95% CI 0.787–0.952, and *P* = 0.003; CC versus GC/GG: OR = 0.835, 95% CI 0.774–0.901, and *P* = 0.000 (Figure 2(a)), and this positive association also was maintained in ethnicity subgroup analysis. 11 out of the 12 included studies were conducted in Asian population. Significant association remained in Asian population in the following genetic models: C versus G: OR = 0.878, 95% CI 0.834–0.925, and *P* = 0.000; CC versus GG: OR = 0.777, 95% CI 0.697–0.867, and *P* = 0.000; GC/CC versus GG: OR = 0.850 95% CI 0.771–0.937, and *P* = 0.001; CC versus GC/GG: OR = 0.834, 95% CI: 0.771–0.901, and *P* = 0.000 (Figure 2(b)).

For miR-196a2 C>T, the results were shown in Table 3. Association between rs11614913 polymorphism and HCC risk was observed in the following models (using the random-effects model): T versus C: OR = 0.891, 95% CI 0.815–0.974, and *P* = 0.011; TT versus CC: OR = 0.783, 95% CI: 0.649–0.943, and *P* = 0.010; CT versus CC: OR = 0.831, 95% CI 0.714–0.967, and *P* = 0.017; CT/TT versus CC: OR = 0.817, 95% CI 0.703–0.949, and *P* = 0.008. The results suggested that miR-196a2 C allele carrier may be susceptible to HCC. In subgroup analysis, there was also significant association in Asian population (using the random-effects model, T versus C: OR = 0.910, 95% CI 0.837–0.990, and *P* = 0.029; TT versus CC: OR = 0.817, 95% CI: 0.684–0.976, and *P* = 0.026; CT versus CC: OR = 0.838, 95% CI 0.712–0.986, and *P* = 0.033; CT/TT versus CC: OR = 0.833, 95% CI 0.712–0.974, and *P* = 0.022).

### 3.3. Heterogeneity, Sensitivity Analysis, and Publication Bias

*Q*-test was used in all of the genetic models to test heterogeneity. For miR-146a G>C, it showed no significant heterogeneity between studies during overall comparisons (Table 2). For miR-196a2 C>T, heterogeneity was observed in all models (Table 3): T versus C: *P*_*h*_ = 0.012 and *I* ^2^ = 57.7%; TT versus CC: *P*_*h*_ = 0.007 and *I* ^2^ = 60.6%; CT versus CC: *P*_*h*_ = 0.025 and *I* ^2^ = 52.7%; TC/TT versus CC: *P*_*h*_ = 0.012 and *I* ^2^ = 57.6%; TT versus CT/CC: *P*_*h*_ = 0.094 and *I* ^2^ = 39.6%. Then, we assessed the source of heterogeneity by ethnicity, source of controls, genotyping methods, publication year, and sample size (subjects > 500 in both cases and controls). Using metaregression analysis, none of them could explain the significant heterogeneity (Table 4). In addition subgroup analysis was performed; substantial heterogeneity still existed when stratified by ethnicity (*P*_*h*_ = 0.007 and *I* ^2^ = 60.6%), source of controls (*P*_*h*_ = 0.009 and *I* ^2^ = 61.0%), and sample size (*P*_*h*_ = 0.023 and *I* ^2^ = 68.5%).

**Figure 3:**
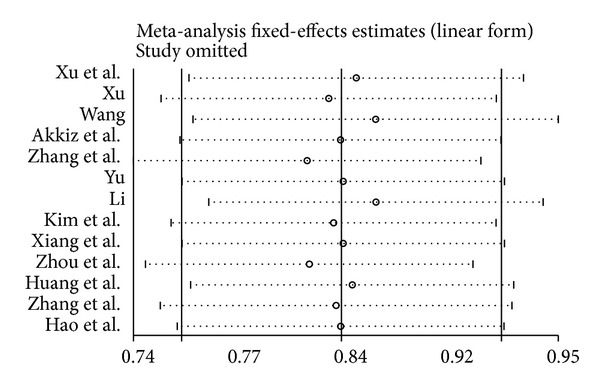
Sensitivity analysis for* mir-146a G>C* polymorphism with hepatocellular carcinoma (CC versus GC/GG).

To assess the influence of each individual study on the pooled ORs, the sensitivity analysis was performed by removing a single study from meta-analysis sequentially. The results indicated that no single study influenced the pooled OR qualitatively (Figure 3). It suggested that the results of this meta-analysis were stable.

**Figure 4:**
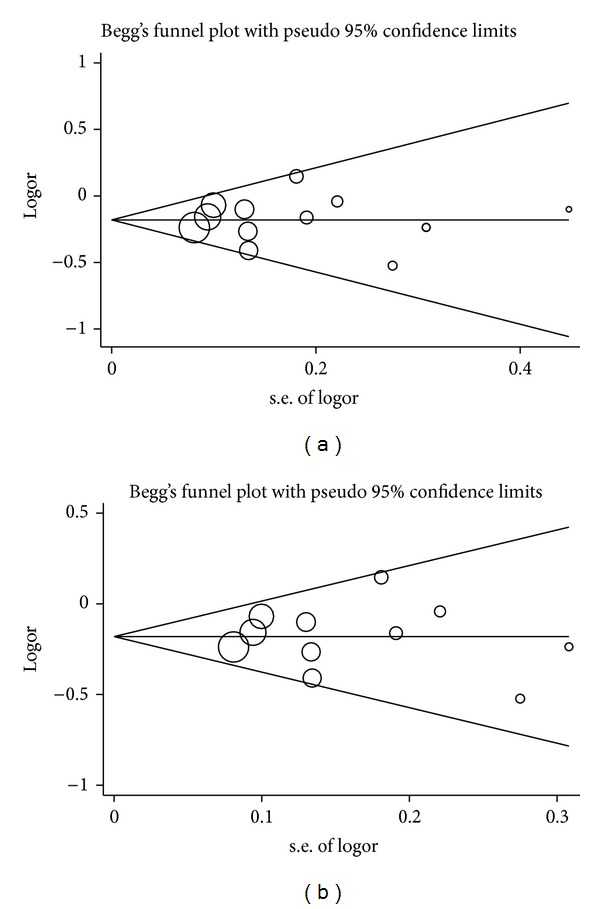
Begg's funnel plot for publication bias test for* mir-146a G>C* polymorphism with hepatocellular carcinoma in overall population (a) and in Asian population (b). s.e.: Standard Error; logor: logOR (logarithms of Odds Ratio).

The Begg funnel plot and Egger's test were conducted to assess publication bias. The shapes of the funnel plots did not reveal any evidence of obvious asymmetry in all comparison models (Figure 4). Then, Egger's test was used to provide statistical evidence of funnel plot symmetry. The results still did not show any evidence of publication bias (*t* = −2.00 and *P* = 0.074 for miR-146a G>C and *t* = 1.18 and *P* = 0.273 for miR-196a2 C>T).

## 4. Discussion

miRNAs are involved in a variety of biological processes and regulate hundreds of gene targets [12]. The study of miRNAs provides a new view of the pathophysiological mechanism of the etiology and development of HCC. SNPs in miRNA sequence have the potential to function as new diagnostic and prognostic biomarkers for high risky population in an early stage [42, 43]. Moreover, the identification of SNPs may lead new sights to personalized therapy and small molecular interventions for liver cancer.

MiR-146a G>C, or rs2910164 polymorphism which locates in the passenger strand of miR-146a, can disturb the secondary structure and maturation of miR-146a [26, 44]. Xu et al. [36] found that target genes of miR-146a, such as tumor necrosis factor receptor-associated factor 6 and interleukin-1 receptor-associated kinase 1, are key adapter molecules downstream of the Toll-like and cytokine receptors in the signaling pathways that play crucial roles in cell growth and immune recognition. So individuals with GG genotype of miR-146a gene have an increased level of mature miR-146a and are more susceptible to carcinogens that promote HCC. Li [32] found miR-146a may target DNA repairing genes such as XRCC1, BRCA11, and XPC. G>C polymorphism can affect mature miR-146a expression and associate with HCC susceptibility. However, in contrast, Akkiz et al. [26] and Kim et al. [30] demonstrated that the rs2910164 polymorphism had no major role in the susceptibility to HCC and they attribute their discrepancy with other studies to ethnic variation in the population. Besides, Zhou et al. [41] indicated polymorphisms of miR-146a were related to the age of onset and Child-Pugh grade in HCC but lacked association with the risk of HCC. Xiang et al. [35] and Zhang et al. [40] also observed no significant difference in ORs of the miRNA-146a variant among HCC patients.

To explain these conflicting results, our meta-analysis, which was based on eleven studies and involved 5689 cases and 6790 controls, was conducted to derive a more precise estimation of the association. Our results suggested that miR-146a G>C polymorphism was associated with decreased risk of hepatocellular carcinoma among the included studies, especially in Asian population. Cochran's *Q*-test and Egger's test showed no significant heterogeneity or publication bias, which indicated that our results were stable. Since miR-146a regulates hundreds of downstream gene targets, it is biologically plausible that rs2910164 polymorphism may alter the oncogenesis genetic pathway and modulate hepatocellular carcinoma risk.

MiR-196a2 C>T polymorphism is another potential SNP in relevance to HCC. It not only affected the maturation of miR-196a2 but also could enhance the cell response to mutagen challenge [31, 45, 46]. Li et al. [31] and Qi et al. [33] found that C allele carriers have a higher incidence of HCC than T allele carriers, which suggested that the C allele may confer risk to the occurrence of HCC. Studies have indicated that high expression of miR-196a2 could deregulate target genes including homeobox (HOX) gene cluster and annexinA1 (ANXA1) gene and lead to carcinogenesis and malignant transformation of HCC [25, 40, 47]. However, Li [32] and Kim et al. [30] observed no significant difference of the TT, TC, and CC genotypes distribution between HCC patients and controls. Han et al.'s study [27] also showed miR-196a2 polymorphism was not statistically associated with HCC risk, though it may enhance the effects of other SNPs in relevance to HCC.

Our meta-analysis included 9 case-control studies to assess the relationship between MiR-196a2 C>T polymorphism and HCC. The results indicated that T allele carriers had significantly lower HCC susceptibility, especially in Asian population. Identification of heterogeneity is one of the most important goals of meta-analysis and heterogeneity existed in our study. However, through subgroup analysis and meta-regression analysis we could not find the source of heterogeneity, which suggested that these included studies may be different in either clinical, methodological, or statistical components and the quantitative synthesis.

There are three similar meta-analyses about the association between the miR-146a G>C polymorphism or the miR-196a2 C>T polymorphism and the risk of hepatocellular carcinoma, but their studies showed different results from ours. Wang et al. 2012 [48] carried out a meta-analysis to estimate the relevance between these two SNPs and HCC susceptibility and it concluded that neither the rs2910164 nor rs11614913 polymorphism was associated with HCC risk. Their results were in the opposite direction to ours possibly due to the relatively small sample size. Their last search update was on September 10, 2012, and they totally identified 6 studies including 1912 cases and 2149 cases for miR-146a G>C polymorphism and 1790 cases and 1635 controls for miR-196a2 C>T polymorphism. In our study we included a total of 17 studies with 5689 cases and 6790 cases for miR-146a G>C polymorphism and 5249 cases and 5177 controls for miR-196a2 C>T polymorphism. Our sample size was much larger and could lead to the difference. Hu et al. 2013 [49] performed a meta-analysis to assess the contributions of the rs2910164 and rs3746444 polymorphisms to HCC susceptibility. Possibly because of the same reason of Wang's, their study showed no significant association. The meta-analysis of Xu et al. 2013 [50] revealed the miR-146a C variant was associated with a decreased HCC risk and it was consistent with ours. Their study only comprised a total of ten case-control studies involving 3437 cases and 3437 controls. We extracted data from all the published studies and added another 7501 cases and 8530 controls to the analysis, which accounted for 69.9% of the total sample size. Thus our results were more precise and persuasive. With regard to rs11614913, they concluded that the miR196a2 T variant was associated with a decreased risk of HCC. However, heterogeneity existed among studies. Our pooled effects were also statistically significant, but we failed to find the source of heterogeneity by subgroup analysis and metaregression analysis. So we concluded that miR-196a2 C>T polymorphism may contribute to a decreased HCC risk, but the results need to be validated by more qualified studies. In our present meta-analysis, we searched multiple databases and included all eligible studies. It contained the newest data and largest sample size. Compared with previous meta-analyses, we generate more exact and powerful pooled results of the association between SNPs miR-146a G>C and miR-196a2 C>T and risk of hepatocellular carcinoma.

There are some limitations in this meta-analysis that must be addressed. First, in the subgroup analyses, only one study originated from Caucasian, the size of which was small, and there was no African population. So our study mainly suggested the association between the two SNPs and HCC susceptibility in Asian population and may not be generalized to other ethnicities. Further studies on other ethnicities are necessary to validate the results. Second, lack of original data like HBV infection status, alcohol consumption, age, and gender from the included studies limited our further stratified analysis. HBV is one the most important risk factors to HCC [51], and the interactions among gene-gene and gene-environment may relate to cancer risk. Insufficient information prevented us from performing further evaluation. Third, heterogeneity was detected in overall comparisons of miR-196a2 C>T and we could not find its source. Though miR-196a2 C allele carrier was shown to have a higher risk of HCC in our study, more studies using standardized unbiased methods and well-matched controls are needed to draw a more persuasive conclusion. Last, as the two miRNAs have some other more SNPs than miR-146a G>C and miR-196a2 C>T, this analysis cannot tell the contribution of other polymorphisms to the risk of hepatocellular carcinoma.

In conclusion, our meta-analysis provided evidence that the two common polymorphisms miR-146a G>C and miR-196a2 C>T were associated with decreased HCC susceptibility, especially in Asian population. Additional well-designed, large studies are warranted to validate our findings and further functional studies should be conducted to elucidate its mechanism. More sufficient data such as hepatitis infection status, gene-environment interactions, and multiethnic groups should be considered in future studies to lead to a more comprehensive understanding of the association between miR-146a G>C and miR-196a2 C>T polymorphisms and the risk of HCC.

## Figures and Tables

**Table 1:** Characteristics of literatures included in the meta-analysis on hepatocellular carcinoma.

Author	Year	Country	Ethnicity	Source of controls	Genotyping method	Cases/controls	MicroRNA polymorphism	Allele frequency G/C or C/T	HWE
Xu et al. [36]	2008	China	Asian	Population	PCR-RFLP	479/504	miR-146a G>C	0.36/0.64	0.119
Xu [37]	2010	China	Asian	Population	PCR-RFLP	500/522	miR-146a G>C	0.39/0.61	0.296
						492/495	miR-196a2 C>T	0.46/0.54	0.621
Qi et al. [33]	2010	China	Asian	Population	PCR-LDR	361/391	miR-196a2 C>T	0.49/0.51	0.869
Li et al. [31]	2010	China	Asian	Hospital	PCR-RFLP	310/222	miR-196a2 C>T	0.42/0.58	0.402
Wang [34]	2011	China	Asian	Population	MALDI-TOF	1116/1869	miR-146a G>C	0.39/0.61	0.115
Akkiz et al. [25]	2011	Turkey	Caucasian	Population	PCR-RFLP	222/222	miR-146a G>C	0.80/0.20	0.384
Akkiz et al. [26]	2011	Turkey	Caucasian	Population	PCR-RFLP	185/185	miR-196a2 C>T	0.55/0.45	0.492
Zhang et al. [39]	2011	China	Asian	Population	PIRA-PCR	925/840	miR-146a G>C	0.41/0.59	0.149
						934/837	miR-196a2 C>T	0.47/0.53	0.972
Yu [38]	2012	China	Asian	Population	PCR-RFLP	100/100	miR-146a G>C	0.44/0.56	0.506
Li [32]	2012	China	Asian	Population	AS-PCR	560/560	miR-146a G>C	0.42/0.58	0.196
						560/560	miR-196a2 C>T	0.39/0.61	0.056
Kim et al. [30]	2012	Korea	Asian	Population	PCR-RFLP	159/201	miR-146a G>C	0.38/0.62	0.190
						159/201	miR-196a2 C>T	0.49/0.51	0.356
Xiang et al. [35]	2012	China	Asian	Population	PCR-RFLP	100/100	miR-146a G>C	0.44/0.56	0.506
Zhou et al. [41]	2012	China	Asian	Population	PCR-RFLP	186/483	miR-146a G>C	0.41/0.59	0.056
Huang et al. [29]	2013	China	Asian	Population	MALDI-TOF	110/110	miR-146a G>C	0.32/0.68	0.122
Zhang et al. [40]	2013	China	Asian	Population	MassArray	997/998	miR-146a G>C	0.39/0.61	0.911
						996/995	miR-196a2 C>T	0.42/0.58	0.245
Han et al. [27]	2013	China	Asian	Population	RT-PCR	1017/1009	miR-196a2 C>T	0.46/0.54	0.310
Hao et al. [28]	2013	China	Asian	Population	PCR-RFLP	235/281	miR-146a G>C	0.38/0.62	0.056
						235/281	miR-196a2 C>T	0.52/0.48	0.051

HWE: Hardy-Weinberg equilibrium; PCR: polymerase chain reaction; RFLP: restriction fragment length polymorphism; PIRA: primer introduced restriction analysis; LDR: ligase detection reaction; AS: allele specific; MALDI-TOF: matrix-assisted laser desorption/ionization time-of-flight.

**Table 2:** Meta-analysis of the miR-146a G>C polymorphism associated with hepatocellular carcinoma.

Comparisons	OR	95% CI	*P*	*P*_*h*_
Overall				
C versus G	0.883	0.839–0.930	0.000	0.230
CC versus GG	0.780	0.700–0.869	0.000	0.167
GC versus GG	0.920	0.832–1.017	0.104	0.199
GC/CC versus GG	0.865	0.787–0.952	0.003	0.125
CC versus GC/GG	0.835	0.774–0.901	0.000	0.545
Asian				
C versus G	0.878	0.834–0.925	0.000	0.255
CC versus GG	0.777	0.697–0.867	0.000	0.129
GC versus GG	0.905	0.816–1.004	0.060	0.216
GC/CC versus GG	0.850	0.771–0.9373	0.001	0.159
CC versus GC/GG	0.834	0.771–0.901	0.000	0.461

*P*_*h*_: *P* value of *Q* test for heterogeneity test; OR odds: odds ratio; CI: confidence interval.

**Table 3:** Meta-analysis of the miR-196a2 C>T polymorphism associated with hepatocellular carcinoma.

Comparisons	OR	95% CI	*P*	*P*_*h*_
Overall				
T versus C	0.891	0.815–0.974	0.011	0.012
TT versus CC	0.783	0.649–0.943	0.010	0.007
CT versus CC	0.831	0.714–0.967	0.017	0.025
CT/TT versus CC	0.817	0.703–0.949	0.008	0.012
TT versus TC/CC	0.907	0.807	1.020	0.094
Asian				
T versus C	0.910	0.837–0.990	0.029	0.036
TT versus CC	0.817	0.684–0.976	0.026	0.021
CT versus CC	0.838	0.712–0.98	0.033	0.017
CT/TT versus CC	0.833	0.712–0.974	0.022	0.012
TT versus TC/CC	0.935	0.846–1.032	0.181	0.270

*P*_*h*_: *P *value of *Q* test for heterogeneity test; OR odds: odds ratio; CI: confidence interval; random-effects model was used when *P*_*h*_≦0.10; otherwise, fix-effects model was used.

**Table 4:** Metaregression analysis for heterogeneity in studies on the miR-196a2 C>T polymorphism associated with hepatocellular carcinoma.

Sort	*P*	*τ* ^2^	*I* ^2^
Ethnicity	0.119	0.0078	51.50%
Source of controls	0.287	0.0097	56.48%
Genotyping method	0.382	0.0134	61.50%
Year	0.976	0.1454	62.34%
Sample size	0.397	0.1194	58.59%
